# The Type of Responder T-Cell Has a Significant Impact in a Human In Vitro Suppression Assay

**DOI:** 10.1371/journal.pone.0015154

**Published:** 2010-12-03

**Authors:** Srikanta Jana, Hope Campbell, Jeffrey Woodliff, Jill Waukau, Parthav Jailwala, Jugal Ghorai, Soumitra Ghosh, Sanja Glisic

**Affiliations:** 1 Department of Pediatrics, Max McGee National Research Center for Juvenile Diabetes and Human and Molecular Genetics Center, Medical College and Children's Hospital of Wisconsin, Milwaukee, Wisconsin, United States of America; 2 Department of Mathematical Sciences, University of Wisconsin, Milwaukee, Wisconsin, United States of America; 3 Blood Research Institute, Milwaukee, Wisconsin, United States of America; 4 Department of Pediatrics, Medical College of Wisconsin, Milwaukee, Wisconsin, United States of America; New York University, United States of America

## Abstract

**Background:**

In type 1 diabetes (T1D), a prototypic autoimmune disease, effector T cells destroy beta cells. Normally, CD4^+^CD25^+high^, or natural regulatory T cells (Tregs), counter this assault. In autoimmunity, the failure to suppress CD4^+^CD25^low^ T cells is important for disease development. However, both Treg dysfunction and hyperactive responder T-cell proliferation contribute to disease.

**Methods/Principal Findings:**

We investigated human CD4^+^CD25^low^ T cells and compared them to CD4^+^CD25^-^ T cells in otherwise equivalent *in vitro* proliferative conditions. We then asked whether these differences in suppression are exacerbated in T1D. In both single and co-culture with Tregs, the CD4^+^CD25^low^ T cells divided more rapidly than CD4^+^CD25^-^ T cells, which manifests as increased proliferation/reduced suppression. Time-course experiments showed that this difference could be explained by higher IL-2 production from CD4+CD25^low^ compared to CD4+CD25- T cells. There was also a significant increase in CD4+CD25^low^ T-cell proliferation compared to CD4+CD25- T cells during suppression assays from RO T1D and at-risk subjects (n = 28, p = 0.015 and p = 0.024 respectively).

**Conclusions/Significance:**

The *in vitro* dual suppression assays proposed here could highlight the impaired sensitivity of certain responder T cells to the suppressive effect of Tregs in human autoimmune diseases.

## Introduction

The immune system of a healthy organism maintains immune balance between tolerance and active response. Under normal physiological conditions, the immune balance is a tightly regulated network of several types of immune cells. If this is perturbed, the response can be either inefficient (as in cancer) or, conversely, over-reactive, resulting in conditions such as autoimmunity. Maintaining peripheral self-tolerance by reducing effector T cell function (suppression) is crucial in preventing autoimmunity. Several mechanisms have been proposed, including induction of T-cell anergy, immunological ignorance and control of the expression of co-stimulatory molecules necessary for activation of naïve, autoreactive T cells. In addition, regulatory T cells impart direct suppression of effector function of autoaggressive T cells [1]. One regulatory subset that has been well characterized, expresses cell-surface IL-2Rα-chains (CD25) constitutively [2]. Adoptive transfer of these CD4^+^CD25^high^ regulatory T cells (Tregs) in animal models has been shown to offer protection from several autoimmune diseases [3], [4], [5]. Thus, Tregs are highly specialized cells that play a pivotal role in the control of autoimmunity.

In human subjects 1–3% of the CD4^+^ T cell population expressing the highest levels of surface CD25 demonstrates regulatory properties [6]. These cells constitutively express CD25 and Foxp3 and show increased expression of CTLA-4, HLA DR, GITR and CD45RO, among other markers, although it has been proven recently that resting nTregs express CD45RA [7]. However, these surface markers are also expressed by activated T cells. Tregs differ from activated T cells by decreased expression of CD127 [8], which somewhat increases confidence in the isolation of Tregs and in their enumeration. Nevertheless, a population of CD4+CD25+CD127^low/-^ T cells does not exhibit enhanced suppressor function compared to CD4+CD25^high^ T cells in our hands (Glisic S, unpublished) as well as in studies of Miyara et al [7]. Therefore, the unique property so far known to distinguish Tregs from non-Tregs is their capacity to suppress proliferation of other T cells. Suppression assays can be performed both *in vivo* [9] and *in vitro*, the latter being the only option for human studies. The *in vitro* assay usually involves responder T cells, antigen-presenting cells (APC) and Tregs. Although our understanding of suppression is increasing [10], the exact relationship between cells in a suppression assay (both *in vivo* and *in vitro*) has not been completely understood. A functional defect of Tregs was reported in many autoimmune diseases (AD): SLE [11], rheumatoid arthritis (RA) [12], multiple sclerosis [13] and type 1 diabetes [14]. However, evidence is emerging that Treg dysfunction cannot fully explain decreased suppression of responder T cell proliferation in AD. Recent reports showed that impaired sensitivity of responder T cells (including autoreactive cells) to suppression by Tregs led to a defective suppression of their proliferation in disease state in NOD mice and human subjects with SLE and T1D [15], [16], [17]. The importance of the presence of antigen-presenting cells (APC) in *in vitro* suppression assay was also recently shown [18]. This report suggested that APCs could be a major source of defects in suppression in T1D. Other reports proved the important role of co-stimulatory ligands on APC (CD28:B7, 4-1BB:4-IBBL, ICOS:CD275, CD40:CD40L) which promote proper stimulation of T cells, and, hence, suppression of responders' proliferation [19], [20], [21]. In addition, the type and strength of stimulation used in an *in vitro* suppression assay is known to affect suppression in both healthy and diseased states [6], [22].

Such variability in the human suppression assay prompted us to perform a comprehensive study investigating the aforementioned factors. After we found the optimal *in vitro* conditions using cells isolated from human leukopacks (enriched leukocytes from whole blood) obtained from anonymous donors, we performed a study involving T1D-related subject groups to test the hypothesis that Tregs cannot suppress two different responder T cell subsets equally well (CD4+CD25- and CD4+CD25^low^). We expected the difference in suppressive potential on the two cell subsets to be amplified in subjects affected with or at risk to develop T1D compared to control subjects.

Results generated in this study suggest that (i) the type of the responder T cells set up in an *in vitro* suppression assay has the greatest impact on suppression by Tregs (ii) CD4^+^CD25^low^ T cells divide more than CD4^+^CD25^-^ T cells and are more difficult to suppress; (iii) the two cell types have different dynamics of IL-2 production, proliferation and death.

## Results

### The type of responder T cell has the greatest impact in a T-cell suppression assay

**Figure 1:**
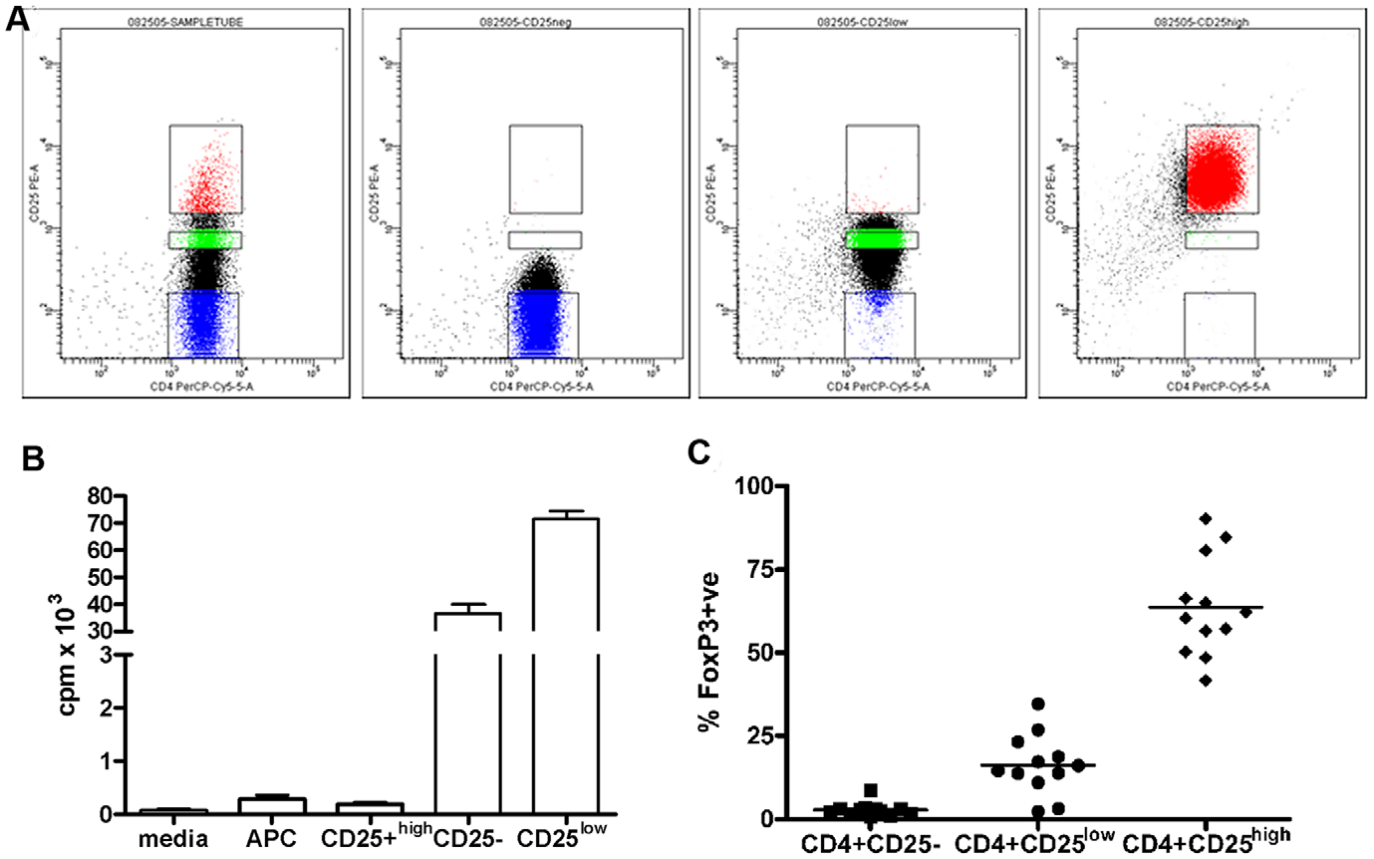
Isolation of human CD4^+^CD25^high^ and CD4^+^CD25^low^ T cells from PBMC. A) Purified human CD4 T cells were stained with PE-Cy5-conjugated anti-human CD4, anti-human CD25-PE and FITC-conjugated anti-human antibodies for CD14, CD32, CD-116 and CD8. CD4^+^CD25^high^, CD4^+^CD25^low^ and CD4^+^CD25^-^ T cells were then isolated excluding large activated lymphocytes and FITC-stained cells. Purity of FACS-isolated three T cell subsets after the isolation is shown. Tregs purity was regularly >97%, while the purity for other two subsets was >94%. B) *In vitro* proliferation of cell cultures presented. Proliferation of CD25^low^ T cells was significantly higher compared to the proliferation of CD25- (t-test, p = 0.003). C) Intracellular Foxp3 staining in three FACS-isolated cell subsets.

**Figure 2:**
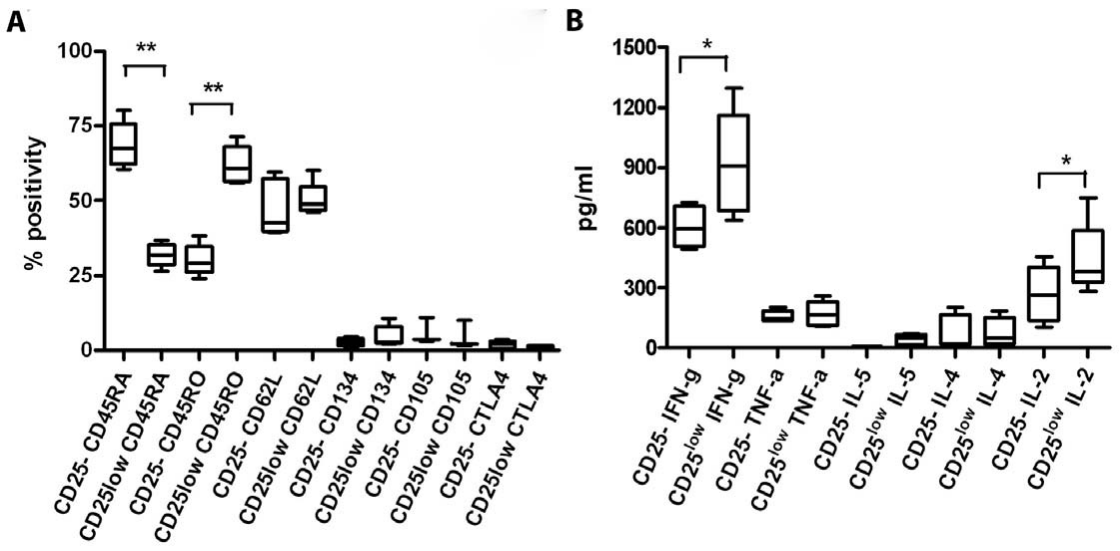
Analysis of surface molecules and cytokine production by sorted T cells. Presented are median values for expression of A) surface molecules and production of B) cytokines in both CD4+CD25- and CD4^+^CD25^low^ T cells. Aliquots were incubated with appropriate flurochorome-tagged antibody for surface markers CD45RA, CD45RO, CD62L, HLA DR, CD69 and HLA DQand analyzed by FACS (n = 6). Statistically significant difference in expression was reached for CD45RA, CD45RO, HLA DR and CD69 (Mann-U-Whitney test, p = 0.0022, p = 0.0022, p = 0.038 and p = 0.0048, respectively). The cytokine production in CD4+CD25- was significantly lower for IL-2 and IFN-γ compared to CD4^+^CD25^low^ T cell production of the same cytokines (p = 0.027 and p = 0.015, respectively, n = 6). CD4^+^CD25^low^ and CD4^+^CD25^-^ T cells were cultured with 1 µg anti-human CD3 beads and 1xirradiated PBMC for 48 hours. Cytokines were measured using CBA assay. * - p<0.05; ** - p<0.005.

**Figure 3:**
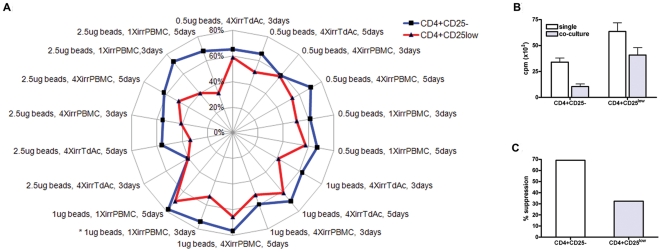
CD4^+^CD25^low^ T cells were suppressed less than CD4^+^CD25- in *in vitro* co-culture with Tregs. A) The percentage of suppression for CD4^+^CD25- (blue, outer line) and CD4^+^CD25^low^ T cells (red, inner line) as responders in co-culture with Treg (Tresponder:Treg _ = _ 1∶1) for 3 or 5 days with 18 different combinations of stimuli. The suppression difference is most statistically significant when the type of responder T cells differ (p<0.003, n = 6) irrespective of type of APC or duration and strength of stimuli. Asterisk (*) indicates conditions chosen for experiments that followed. B) Presented are cpm and standard errors for conditions with asterisk in Figure 3A. C) Percentages of suppression for conditions shown with asterix in Figure 3A are presented.

CD4^+^CD25^-^, CD4^+^CD25^low^ and CD4^+^CD25^high^ T cells were isolated from human PBMC by flow sorting (Figure 1A). Using FACS, the purity of these isolated cells was analyzed further for CD25 expression (Figure 1A), proliferation (Figure 1B) and intracellular Foxp3 expression (Figure 1C). Foxp3 is usually used as a marker for recognition of Tregs, however, we measured its intracellular presence in all three cell types. FACS-isolated CD4+CD25- and CD4+CD25^low^ were treated further as separate responder T cell subsets. We looked at the expression of different surface markers and measured cytokines produced by the two types of responder T cells (Figs. 2A and 2B). Most of the sorted CD4^+^CD25^low^ T cells showed activated phenotype, significantly lower expression of CD45RA (Mann-U-Whitney test, p = 0.0022) and significantly higher expression of CD45RO (p = 0.002) (Figure 2A). In addition, significantly more CD4^+^CD25^low^ T cells expressed HLA DR and CD69 compared to CD4+CD25- T cells (Mann-U-Whitney test, p = 0.038 and p = 0.0048, respectively). Expression of HLA DQ showed borderline significance also in favor of CD4+CD25^low^ compared to CD4+CD25- T cells (p = 0.057). CD4^+^CD25^low^ T cells produced significantly more IFN-gamma and IL-2 compared to CD4+CD25- T cells even after mild stimulation provided by anti-human CD3-coated beads (1 µg/ml) (Mann-U-Whitney test, p = 0.015 and p = 0.027, respectively, Figure 2B), as well as for IL-10 (data not shown). Both CD4^+^CD25^-^ and CD4^+^CD25^low^ responder T cells were cultured either in the presence or absence of Tregs following 18 separate stimuli (Table 1). The percentage of suppression, averaged over six individual measurements, was plotted as a radar chart to accommodate all the data points (Figure 3A). The percentage of suppression increases with the distance from the center. Using a generalized linear model (GLM), neither the type or number of APC nor time in culture were shown to influence the extent of suppression, even though the strength of TCR stimulation almost reached statistical significance (p = 0.057). However, the difference in suppression between CD25- and CD25^low^ was highly significant, indicating that the type of responder T cell was found to be the major predictor variable for percentage of suppression (p = 0.003, n = 6 subjects, by GLM).

**Table 1:** Tested variables in *in vitro* human suppression assay.

	APC	Strength of stimulation	Time in culture
CD4+CD25- or CD4+CD25^low^	1xPBMC	0.5 µg.ml anti-human CD3-coated beads	3 days
			5 days
		1 µg.ml anti-human CD3-coated beads	3 days
			5 days
		2.5 µg.ml anti-human CD3-coated beads	3 days
			5 days
	4xPBMC	0.5 µg.ml anti-human CD3-coated beads	3 days
			5 days
		1 µg.ml anti-human CD3-coated beads	3 days
			5 days
		2.5 µg.ml anti-human CD3-coated beads	3 days
			5 days
	4xTdAC	0.5 µg.ml anti-human CD3-coated beads	3 days
			5 days
		1 µg.ml anti-human CD3-coated beads	3 days
			5 days
		2.5 µg.ml anti-human CD3-coated beads	3 days
			5 days

Assays with CD4^+^CD25^low^ T cells as responders generally gave lower suppression values than assays with CD4^+^CD25^-^ T cells (radar chart, Figure 3A). This highlights an intrinsic difference between the two types of responder T cells. For subsequent experiments, we used 1 µg anti-human CD3-coated beads, 1 x irrPBMC and cultures cells for 3 days (* in Figure 3A). Although few other conditions gave a substantial difference in suppression between the two responder T cell subsets as well, the chosen conditions were considered optimal as both responder T cell subsets show the most consistent suppression results with the lowest s.e.m. amongst the 18 different treatments for each of the responder T cell subsets. The corresponding cpm values for these specific optimal conditions are presented in Figure 3B and corresponding percentage of suppression in Figure 3C. In addition, this combination of stimuli was used for T cells isolated from both healthy control and T1D-related subject groups (Figure 6).

### CD4^+^CD25^low^ T cells proliferate more than CD4^+^CD25^-^ T cells

**Figure 4:**
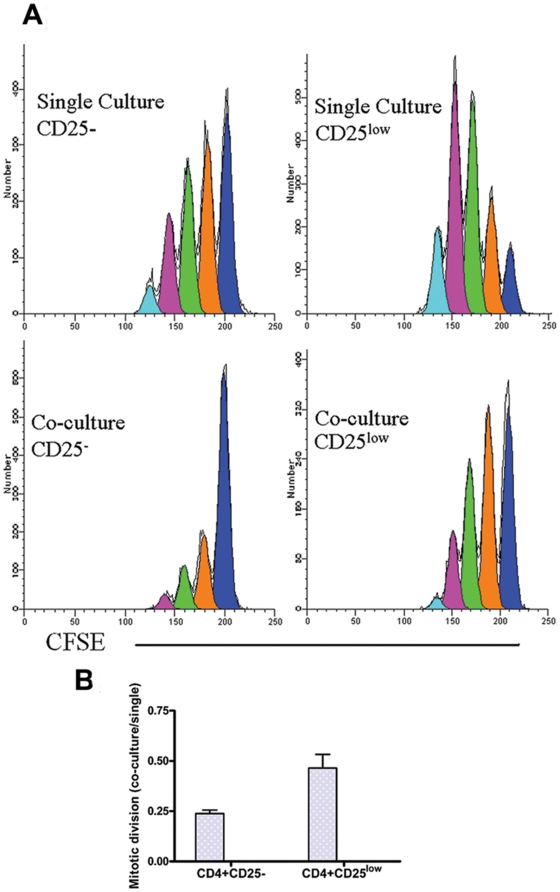
CD4^+^CD25^low^ responder T cells proliferate faster and are harder to suppress. A) CFSE-labeled responder T cells were cultured for 3 days with 1 µg/ml anti-human CD3-coated beads and 1x irradiated PBMC either in the presence or absence of an equal number of Tregs. Each color represents a particular cell generation. Blue color (first peak from right on all four histograms) presents undivided cells. Orange color presents cells that divided once, green peak cells that divided twice, purple color cells that divided three times and light blue presents cells that divided four times. This is representative of three independent experiments. B) The ratio of the normalized mean number of division in co-culture/single culture was calculated using the software ModFit and method of De Boer and Perelson (see Statistical analysis). The average ratio of mitotic divisions for three samples in co-culture versus single culture is 0.23±0.05 for CD4^+^CD25^-^ T cells and 0.46±0.06 for CD4^+^CD25^low^ T cells (p<0.05, n = 3).

The proliferation profiles of the CD4^+^CD25^-^ and CD4^+^CD25^low^ responders were assessed both in single and co-culture. Responder T cells were labeled with CFSE and cultured alone and with Tregs in the presence of 1 µg anti-human CD3 beads and 1xirrPBMC for 3 days. CFSE-labeled responder T cells were gated from other cells in the culture and analyzed for cell division. We found that CD4^+^CD25^low^ responder T cells divided more than CD4^+^CD25^-^ T cells in both single culture and co-culture (Figure 4A). The normalized mean number of divisions (40) for CD4^+^CD25^-^ T cells in single and co-culture were 0.73±0.19 and 0.16±0.04 respectively, whereas for CD4^+^CD25^low^ T cells the corresponding values were 1.12±0.2 and 0.5±0.07. These results show that CD4^+^CD25^low^ T cells divided ∼1.5 times more in single culture and 3 times more in co-culture compared to CD4^+^CD25^-^ T cells. The ratio of mitotic divisions in co-culture versus single culture is 0.23±0.05 for CD4^+^CD25^-^ T cells and 0.46±0.06 for CD4^+^CD25^low^ T cells (Figure 4B, p<0.05, n = 3).

### Different kinetics of IL-2 production in CD4+CD25- and CD4+CD25^low^ T cells

**Figure 5:**
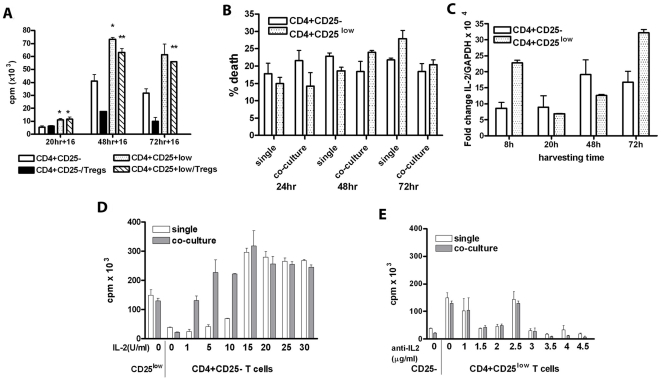
Time-course experiment showed different dynamic of proliferation, death and IL2-production in CD4+CD25- and CD4+CD25^low^ T-cells. A) CD4+CD25^low^ proliferated more rapidly than CD4+CD25- T cells. At indicated bars (* and **) both single and co-cultures of CD4+CD25^low^ had significantly higher cpm (*p<0.05 and ** p<0.01, respectively) compared to CD4+CD25- T-cell single and co-cultures. B) Single and co-cultures were harvested, washed and stained with 7AAD before acquiring. CD4+CD25- T cells were dying at a slightly faster rate at the earlier time points in both single and co-cultures versus at later time points. Mean values of three samples are presented. Borderline significance was detected between the two responders for their co-cultures after 24 hours time point and for single cultures at 72 hours (p = 0.05 and p = 0.08, respectively). C) CFSE-labeled responder T cells were stimulated, harvested, FACS sorted as CFSE-positive cells and stored in Trizol at −80°C without APC and other cells present in cultures, until RNA was isolated and real-time RT-PCR performed. IL-2 production was higher in CD4+CD25^low^ T cells at earlier time point (8 hours), compared to CD4+CD25- T cells. However, at later time points (20 and 48 hours), CD4+CD25- showed higher IL-2 production compared to CD4+CD25^low^ T cells, which switched back again to CD4+CD25^low^ T cells at 72 hours. Mean values of three samples are presented. D) Addition of 20 IU/ml of recombinant IL-2 to CD4+CD25- T cells cultures caused similar ratio single/co-culture proliferation as detected in CD4+CD25^low^ T-cells after 3 days of *in vitro* stimulation (CD4+CD25^low^ are presented in the first column for comparison). This is one of 2 separate experiments with similar results. E) Addition of 3.5 µg/ml of neutralizing, anti-human IL-2 was needed in CD4+CD25^low^ cultures to cause similar ratio of single/co-culture proliferation as the one recorded in CD4+CD25- T cells (CD4+CD25- are presented in the first column for comparison). This is one of 2 separate experiments with similar results.

Counts per minute at three different time points were used to record the distinct proliferation of the two responder T-cell subsets on the stimulation. CD4+CD25- T cells proliferated significantly less compared to CD4+CD25^low^ T cells at all time points (p<0.05 at 24 hours and p<0.01 at later time points, Figure 5A). We also measured % of dead cells in single and co-culture at three time points during culturing of both responder T cell subsets (Figure 5B). CD4+CD25- were dying at slightly higher rate in single culture compared to CD4+CD25^low^ T cells during the first 48 hours, which changed at 72 hours. In co-culture, the faster death rate of CD4+CD25- T cells at 24 hours compared to CD4+CD25^low^ flipped at 48 and 72 hours, when CD4+CD25^low^ started dying faster (Figure 5B). Since it has been reported that IL-2 is directly involved in the proliferation of responder T cells [23] and that human Tregs are dependent on available IL-2 for their suppressor activity [24], we monitored IL-2 production in both responder T-cell subsets at 4 different time points. This was done using RT-PCR (Figure 5C). CD4+CD25^low^ T cells responded by transcribing IL-2 mRNA faster/sooner than CD4+CD25- T cells. At 8 hours CD4+CD25^low^ T cells showed >2 fold increase in IL-2 mRNA production compared to CD4+CD25- T cells (t-test, p = 0.02). At 20 and 48 hours, IL-2 mRNA production in CD4+CD25- was higher than in CD4+CD25^low^ T cells at the same time points. However, at the last measured time point (72 hours), CD4+CD25^low^ T cells increased IL-2 expression almost 2 fold compared to CD4+CD25- T cells reaching significance again (t-test, p = 0.038). This can be explained by bimodal pattern of IL-2 expression in CD4+CD25^low^ T cells. Namely, CD4+CD25^low^ T cells express IL-2 sooner, proliferate faster, and start dying later. CD4+CD25^low^ T cells that survive begin rapid IL-2 production and proliferation, while CD4+CD25- T cells responded at a slower rate. Furthermore, proliferation and suppression of both T cell responder subsets under different concentrations of recombinant IL-2 or anti-human IL-2 was also recorded. Addition of 20 IU/ml of recombinant IL-2 to both single and co-culture of CD4+CD25- responder T cells was able to produce the same proliferation ratio between single and co-culture as seen in CD4+CD25^low^ T cells without exogenous IL-2 (Figure 5D). When single and co-cultured CD4+CD25^low^ T cells were treated with different concentrations of anti-human IL-2, the proliferation ratio, seen in single and co-cultured CD4+CD25-, was achieved with 3 µg/ml of anti-human IL-2 (Figure 5E). Thus, we were able to reconstitute the observed proliferation differences between single culture and co-cultures for the alternative responder T cells by addition of recombinant IL-2 to CD25- assays or by deprivation of IL-2 in CD25^low^ assays. These experiments confirm existing differences in kinetics of IL-2 production between CD4+CD25- and CD4+CD25^low^ T cells. It should be noted that experimental conditions were slightly changed because of potential changes in viability. Thus, in this series of experiments with addition of IL-2 and anti-IL-2 cells were in culture 54 hours, pulsed and left in culture for additional 15 hours (totaling 70 hours).

### CD4+CD25^low^ T cells are harder to suppress in T1D-related subjects

**Figure 6:**
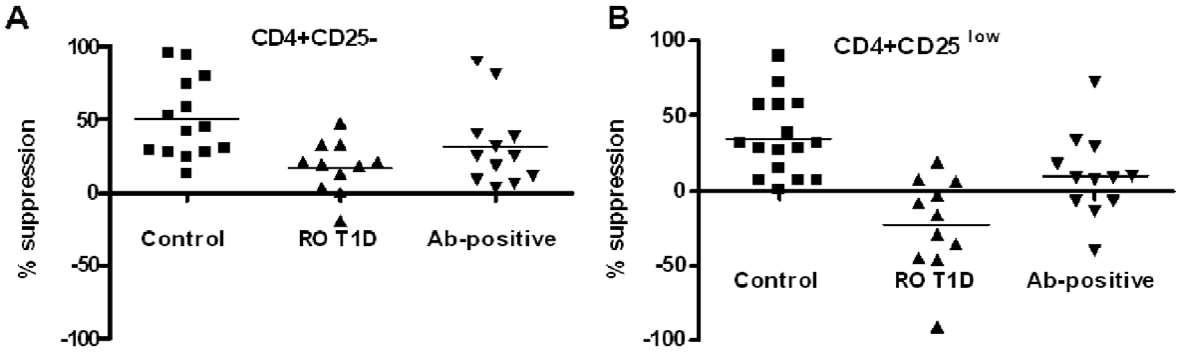
RO-T1D and aAb+ve unaffected subjects show dramatic decrease in suppression of CD4+CD25^low^-Tcell proliferation by Tregs. There was statistically significant difference in suppressive potential of CD4+CD25- T cells between control, RO T1D and aAb+ve subjects (Kruskal-Wallis test, p = 0.014, Figure 6A). However, the difference was even more pronounced with CD4+CD25^low^ as responder T cells (Kruskal-Wallis test, p = 0.0007, Figure 6B). Both RO T1D and autoantibody-positive subjects showed significant decrease in suppression of CD4+CD25^low^ T cells (paired t-test, p = 0.0025 and p = 0.008) compared with their respective CD4+CD25- T cells. In addition, there was significant difference in suppression of the two responder T cells in healthy control subjects (p = 0.017).

The optimal suppression assay (asterix in Figure 3) was then applied to our cohort population. The only difference from the assay developed on anonymous leukopacks was the ratio between Tregs and responders, which we further optimized (Figure S1) due to limited number of available FACS-isolated Tregs from pediatric subjects. The optimal ratio was determined to be 1∶10. This ratio was then used for measurement of Treg function across subject groups. Such high ratio additionally indicated purity of the Treg subset. There was statistically significant difference in suppressive potential of CD4+CD25- T cells between control, recent-onset (RO) T1D and aAb+ve subjects (Kruskal-Wallis test, p = 0.012). However, the difference was even more pronounced for CD4+CD25^low^ T cells (Kruskal-Wallis test, p = 0.0001). In healthy control subjects (n = 14), Tregs showed lower suppressive potential of CD4+CD25^low^ compared to CD4+CD25- T cells (37.6±7.2 vs 50.2±7.3%, p = 0.017, Figure 6B vs. 6A), corroborating results generated with leukopacks. The difference in suppression of CD4+CD25^low^ and CD4+CD25- was also significantly differed in both RO T1D subjects (−22.2±9.6 vs. 17.8±5.5, respectively, p = 0.0025, n = 11, Figure 6B vs 6A) and in aAb-positive subjects (9.9±8.0 vs. 32.0±8.1%, respectively, p = 0.008, n = 12, Figure 6B vs 6A). Control of CD4+CD25^low^ T cells proliferation was, therefore, decreased in all three groups, but, seriously compromised only in RO T1D subjects (Kruskal-Wallis test, p = 0.0001, Figure 6B).

## Discussion

In organ-specific autoimmune disease, the affected organ is largely infiltrated with T lymphocytes by specific homing. At the site, further activation, proliferation, survival and effector function of autoreactive T cells results in tissue damage. It is likely that the autoreactive T cells belong to CD4^+^CD25^low^ T cell population. Tregs are known to regulate these activated T cells, thereby inhibiting their effector function. Although both *in vitro* and *in vivo* regulation of these autoreactive T cells by Tregs has been studied in animal models for many autoimmune diseases, fewer human *in vitro* studies have been performed. Studies exploring CD4^+^CD25^low^ T cells as responders in human *in vitro* suppression assays are scarce [25], [26]. The majority of studies were performed with either naïve CD4^+^CD25^-^ T cells or PBMCs as responders. It has been acknowledged that difference between naïve and activated cells exist, as several studies have shown that CD4^+^CD25^low^ T cells have a different activation status than *in vitro*-activated naïve T cells [27], [28], [29], [30], [31]. Naive T cells, once activated, proliferate and differentiate into effector T cells that differ from naive cells with respect to faster kinetics of their response to re-stimulation. In addition, activated cells are less dependent on co-stimulation in exerting their effector function (cytokine production, migration, proliferation) [32]. In the present study we set out to explore *in-vitro* suppression exercised by FACS-isolated Tregs on two types of human responder T cells: naïve, CD4+CD25- and *in vivo* activated, CD4+CD25^low^ T cells.

The strength of stimuli in co-culture with Tregs dictates whether suppression will occur or not. With high TCR stimulation, suppression is either low or abrogated [33]. When the 18 different stimuli were examined, irrespective of the strength of TCR signal or number and types of irradiated APC cells used in co-culture, the strongest statistical difference in suppression we found to be explained by the type of responder T cells (p<0.003). The strength of TCR signal used in this study had some, rather weak, effect on suppression (p<0.057). Based on these results, we can conclude that the type (nature) of the responder T cells has the greatest impact on suppression assay.

Maintenance of T cell homeostasis is critical in the well-orchestrated immune system and Tregs are one of the key players. They depend on IL-2 produced by other T cells for their development, proliferation and efficient function [34]. One of the indicators of suppression by Tregs is the inhibition of transcription of this proliferative cytokine in responder T cells [35]. Differential suppression of CD4^+^CD25^-^ and CD4^+^CD25^low^ responders by the same Tregs could be due to an unequal inhibition of IL-2 transcript in the two responder T cells. As CD4+CD25^low^ T cells have already been activated, they react faster than naïve T cells on the same stimulus. Our results showed that CD4^+^CD25^-^ and CD4^+^CD25^low^ responder T cells produced different amounts of IL-2 mRNA as well as IL-4 (data not shown). *In vitro* stimulated, CFSE-positive CD4^+^CD25^low^ responder T cells began IL-2 production earlier than CD25-, suggesting different kinetics between the two responder T cells [32]. IL-2 production decreased up to 48 hours in culture, while death rate increased (Figure 5). However, at 72 hours in culture, CD4^+^CD25^low^ responder T cells that survived began a new cycle of IL-2 production and proliferation. This could be explained by bimodal IL-2 expression hypothesis [36]. Bimodal IL-2 expression could be achieved through positive feedback loops of upregulated IL-2R alfa [37], which is differently expressed in CD4+CD25- and CD4+CD25^low^ T cells. In addition, it has been reported that NFATc2 transcription factor, shown as powerful molecular switch of IL-2 expression, shows a bimodal expression [38].

Another possible explanation for reduced suppression of CD4^+^CD25^low^ responder T cells is that with further activation, the pre-activation status of these cells make them divide more easily compared to CD4^+^CD25^-^ T cells. The proliferation profile of CFSE-labeled responder T cells under the same stimuli (Figure 4) showed that CD4^+^CD25^low^ T cells divide significantly more in co-culture than CD4^+^CD25^-^ T cells. The increased proliferation of CD4^+^CD25^low^ T cells may play a role *in vivo* and explain the existence of this expanded T-cell subset in autoimmune disease patients [39], [40]. Several autoimmune patient studies showed that CD4^+^CD25^low^ T cells expressed increased levels of several surface molecules like CD95 [41], CD71 [42], [43], CD26 [27], CD29 [43] and CD28 [44]. These studies clearly indicate that CD4^+^CD25^low^ T cells from autoimmune patients are different from *in vitro* activated naïve-T cells and emphasize the importance of using *in vivo* activated T cells (CD4^+^CD25^low^) as responders in *in vitro* suppression studies. Our results presented in Figure 6 show significant decrease in control of CD4+CD25- T cell proliferation by Tregs isolated from RO T1D subjects, as we showed earlier [45]. In the present study we show that this decrease was even more prominent with CD4+CD25^low^ T cells as responders. In aAb-positive subjects, there was a clear difference in suppressive potential of CD4+CD25- compared to CD4^+^CD25^low^ T cells, which could offer an explanation for impaired sensitivity of responder T cells to the suppressive effect of Tregs in autoimmune diseases reported by increasing number of studies [15], [16], [17], [46]. The role of IL-2 is essential for Treg function. Therefore the local cytokine milieu that depends on the activation status of effector T cells, has crucial role on Treg function. Here we showed that our optimized *in vitro* suppression assay, if set up with both naïve and *in vivo* activated T cells as responders, can identify inability of Tregs to control activated T cell proliferation before symptoms of autoimmune disease appear.

Although the generated results were consistent, it should be noted that the isolation of Tregs was performed based on CD25 expression, a surface marker that is not unique to Tregs. However, there is no unique human surface marker to date that would distinguish Tregs from non-regulatory T cells. FACS isolation based on CD25 and CD127 (and especially solely based on CD127) did not show higher suppressive potential in our hands compared to CD4CD25^high^ T cells (data not shown). Other groups have also reported on the unclear advantage of isolation based on CD127/CD25 compared to the use of CD25 [47], [48], [49]. Despite the impurity of Tregs, our results show divergence between the two responder cells, clearly pointing to differences in the two types of cellular interaction that occurs in co-culture.

In summary, based on extensive study presented here, we concluded that human *in vivo* activated T cells and naïve T cells behave differently in *in vitro* suppression assays when set up with natural Tregs. The type of responder cells is an important variable of suppression assay, which can explain resistance to regulatory T cell-mediated suppression noticed by several groups. The increased production of IL-2 by CD4^+^CD25^low^ T cells might explain the observed differences in suppression. We show that the use of this *in vitro* suppression assay with both naïve and *in vivo-*activated T cells as responders is an excellent tool in the recognition of an early state in immune imbalance. Further investigation into the pleiotropic nature of suppression with different responder T cells in T1D and other autoimmune diseases may give more insight into Tregs function in diseased conditions.

## Materials and Methods

### Media, reagents and antibodies

Anti-human CD4-PE-Cy5, anti-human CD25-PE (M-A251), anti-human CD14-FITC, anti-human CD32-FITC, anti-humanCD116-FITC, anti-human CD8-FITC, anti-human CD28 (CD28.2) and Human Th1/Th2 cytokine cytometric bead array (CBA) kit were purchased from BD Bioscience, CA. L-glutamine, sodium pyruvate, HEPES, penicillin/streptomycin were purchased from Life Technologies, MD. Anti-human CD3 (UCHT1) was purchased from Ancell, MN. Anti-human CD4 microbeads and anti-human CD2-magnetic beads were purchased from Miltenyl Biotech, CA. Tosylactivated magnetic beads used for coating with anti-human CD3 were purchased from Dynal Biotech (Norway) and the procedure was done according to manufacturer's protocol using 0.5 µg, 1 µg and 2.5 µg anti-human CD3 per 10^7^ beads for coating. Human AB serum was purchased from Atlanta Biologicals, GA. RPMI 1640 was purchased from BioWhittaker, MD. Human recombinant IL-2 was purchased from BD Biosciences and anti-human IL-2 from R&D Systems.

### Cell isolation

PBMC were isolated from human leukopacks (n = 6) kindly provided by the Blood Center of Wisconsin, Milwaukee by Ficoll-Hypaque (Amersham Pharmacia Biotech, NJ) density gradient centrifugation. The CD4+ T cells were isolated from 9–10×10^8^ PBMC by MACS sorting using anti-human CD4 microbeads. The isolated CD4+ (1–2×10^8^) T cells were stained for FACS isolation as decribed earlier [45]. PBMC from T1D-related subject groups did not undergo MACS sorting, they were directly stained for FACS isolation. Gates for CD4^+^CD25^low^ were made based on Fluorochrome Minus One. Gating and purity of isolated cells is presented in Figure 1a. T cell-depleted accessory cells (TdAc) were isolated by negative selection of PBMC incubated with anti-human CD2-coated beads (Miltenyl Biotech, CA) followed by MACS sorting.

### Human subjects

Thirty seven subjects were ascertained primarily through the diabetes clinic at Children's Hospital of Wisconsin (CHW). Inclusion criteria for control subjects were a random blood glucose <110 mg/dl, no personal and family history of type 1 diabetes and an absence of diabetes-specific autoantibodies (to GAD, insulin and IA-2). Diabetes was defined according to accepted criteria: 1. Symptoms of diabetes plus casual plasma glucose concentration 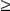
200 mg/dl (11.1 mmol/l) OR 2. FPG 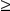
126 mg/dl (7.0 mmol/l). The protocol was approved by the CHW institutional review board (IRB) and participants and/or their parents (guardians) provided written informed consent and completed a questionnaire. All subjects had T1D-specific autoantibodies measured at the recruitment visit. (Table S1 presents the demographic data).

### Cell culture

Both CD4^+^CD25^-^ and CD4^+^CD25^low^ (10^4^ cells/well) were cultured in RPMI 1640 media supplemented with 2 mM L-glutamine, 5 mM HEPES, 100 U/ml penicillin/streptomycin, 0.5 mM sodium pyruvate and 10% human AB serum. Cells were stimulated with anti-human CD3 coated beads (0.5, 1, 2.5 µg, 3 beads/cell) in U-bottom 96 well plates (Costar, NY) in the presence of three variations of autologous irradiated APC: 4 x PBMC, 4 x TdAc or 1 x PBMC for 3 or 5 days. Irradiation was performed at dose of 5000rad. For the suppression assays, Treg cells were co-cultured with CD4^+^CD25^-^ or CD4^+^CD25^low^ at a 1∶1 ratio (Treg:Tresponder). At the end of the culture (3 or 5 days), cells were pulsed with 1 µCi of [^3^H]thymidine (Amersham Pharmacia Biotech, NJ) and harvested after 16 hours. The cpms per well were determined using a scintillation counter (Top Count NXT, Packard, CT). However, in experiments with added IL-2 and anti-IL-2, cells were culture for 54 hours (because of anticipated changes in viability), pulsed and cultured for another 16 hours.

### Carboxyfluoroscein Succinimidyl Ester (CFSE) Staining

CFSE is a vital stain which, upon entering cells, undergoes esterase cleavage and diffuses throughout the cytoplasm. As cells divide, the CFSE is split equally between the daughter cells resulting in diminished CFSE signal detection in flow-cytometric analysis. CFSE was added at a final concentration of 125 nM to 1–2×10^6^/ml of responder T cells and incubated for 15 minutes at room temperature in the dark. The reaction was stopped by adding an equal volume of cold media containing 10% serum and the cells were washed twice with the same media. CFSE-labeled responder T cells were only analyzed for 3 days since by 5 days fractions of responders could not be distinguished from unstained cells. Analysis of divisions of responder T cells was done using the ModFit software (Verity Software House, USA).

### Mortality assay

After 3 days of culture, cells were washed and stained with 7-aminoactinomycin D (7AAD) and analyzed by Flow Cytometry. The population stained with 7AAD has been found to correspond to dead cells. Responder cells in both single and co-culture were assessed for death. The percentage of increase in death in co-culture is X =  [Ac-As]/As x 100, where As  =  dead cells in single culture, Ac  =  dead cells in co-culture.

### Flow cytometry

For all flow-cytometric analyses, cells were stained with the appropriate combination of monoclonal antibodies in PBS containing 0.5% BSA and 2 mM EDTA for 20 minutes at 4°C. After washing, the labeled cells were analyzed or fixed with 2% paraformaldehyde, then analyzed using a FACS Calibur with CellQuest or FACSDIVA software. Cytokines were measured from cell culture supernatants after 48 hours of stimulation (1 µg anti-human CD3 beads and 1 x irrPBMC) with CBA assay kit according to manufacturer's protocol (BD Bioscience). Foxp3 expression was measured by Flow Cytometry using intracellular Foxp3 staining kit from eBioscience, USA.

### RT–PCR

Total cellular RNA was extracted from the sorted CFSE-labeled responder cells after 24, 48 and 72 hours in culture with Trizol reagent. Total RNA was processed as described earlier [50]. The primer sequences were as follows; IL-2: 5′-CAGTGCACCTACTTCAAGTTCTACA-3′ and 5′-CCTGGTGAGTTTGGGATTCTTGTAA-3′. The fold change of IL-2 mRNA has been calculated using the following formula. Fold Change  = 2^-ΔCT^, where ΔCT  =  CT^IL-2^ -CT^GAPDH^. Percentage reduction in IL-2 mRNA expression from single to co-culture is [(Xs-Xc)/Xs] x 100, where Xs  =  fold change of IL-2 in single culture, Xc  =  fold change of IL-2 in co-culture.

### Time-course experiment

Separate plates were set up with CFSE-stained both CD4+CD25- and CD4+CD25^low^ T cells as responders that were isolated from additional leukopacks. Cells were collected at 24+16 hours, 48+16 hours and 72+16 hours, stained with 7AAD, acquired for analysis and the rest was sorted as CFSE+ve cells on FACSVantage or FACS Aria to eliminate Tregs, irradiated PBMC (5000 rad) and anti-human CD3-coated beads. FACS-sorted responder T cells were put in Trizol and kept at -80°C until RNA isolation was performed. At each time point, supernatants were taken out from wells after which they were pulsed for measuring of 3H-thymidine incorporation. Supernatants were kept at -80°C until cytokine measurement using CBA assay was performed.

### Cytokine measurement

Measurement of cytokine levels was performed using the Cytometric Bead Array (CBA) Human Th1/Th2 Cytokine Kit (BD Biosciences). Supernatants were thawed and 50 µl was used for simultaneous measurement of following cytokines: INF-g, TNF-a, IL-5, IL-4 and IL-2. The assay was performed according to the manufacturer's protocol. The samples were acquired on a FACSCalibur (BD Biosciences) following the cytometer setup protocol. The FACS data was analyzed using the CBA 6 bead analysis software (BD Biosciences).

### Statistical analysis

We employed a generalized linear model (GLM) with the dependent variable being the percentage suppression [(s-c)/s] x 100%, where s = cpm in single culture and c = cpm in co-culture. The factors were strength of stimulation (three levels, 0.5, 1 and 2 µg/ml anti-CD3 coated beads), type and amount of irradiated APC (1X PBMC, 4X PBMC and 4X TdAC), days of co-culture (3 or 5 days) and type of responder T cells (CD4^+^CD25^-^ or CD4^+^CD25^low^ T cells). An analysis of variance was performed.

From the CFSE data the numbers of cells in each generation were calculated using the software ModFit. The normalized mean number of division that cells have undergone was calculated using method of De Boer and Perelson, where *X_n_*(*t*)  =  the number of cells undergone *n* divisions by time *t* [51]. Since after *n* divisions, each cell gave rise to a maximum 2*^n^* progeny under ideal conditions, 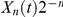 =  (is) the number of precursors that would have generated *X_n_*(*t*) cells. Total number of cell division that *X_n_*(*t*) cells have undergone is expressed as *n* 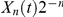. So, the number of precursors that would have generated all the current cell population is 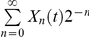 and the total number of cell divisions for the entire current cell population is 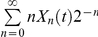. The normalized mean number of division cells have undergone is hence, 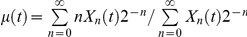.

## Supporting Information

Figure S1 **Titration of responder T cells in suppression assay.** CD4+CD25- were FACS isolated from leukopacks drawn from healthy volunteers and cells were stimulated with 1 µg/ml anti-human CD3 with 1x irradiated PBMC as APC for 3 days. Since extent of suppression began decreasing at 1∶16 ratio, 1∶10 was consistently performed in experiments with T1D-relevant subject groups.
(TIF)

Table S1 Demographic data for T1D-related subject groups
(DOC)
