# Supplementary material for: The Type of Responder T-Cell Has a Significant Impact in a Human In Vitro Suppression Assay
Source: PLoS One. 2010 Dec 3;5(12):e15154. doi: 10.1371/journal.pone.0015154 (PMC2997082; doi:10.1371/journal.pone.0015154)
Supplement: Table S1 — Demographic data for T1D-related subject groups (DOC) [file pone.0015154.s002.doc]

**Table S1.**  Demographic data for T1D-related subject groups

|  | **Control subjects** | | **RO T1D** | **subjects** | **Ab+ve subjects** | |
| --- | --- | --- | --- | --- | --- | --- |
|  |  |  |  |  |  |  |
| **n** | **11** |  | **11** |  | **6** |  |
|  |  |  |  |  |  |  |
| **average time after diagnosis (months)** | **N/A** |  | **0.46** | **± 0.21** | **N/A** |  |
|  |  |  |  |  |  |  |
| **Gender (% Female)** | **40.0** |  | **55.0** |  | **66.0** |  |
|  |  |  |  |  |  |  |
| **Autoantibodies (GAD_65_, IAA, IA2) (%)** | **0.0** |  | **45.0** |  | **0.0** |  |
|  |  |  |  |  |  |  |
| **BMI at recruitment** | **23.03** | **± 1.73** | **19.52** | **± 1.31** | **19.25** | **± 0.83** |
|  |  |  |  |  |  |  |
| **age of diagnosis (yr)** | **N/A** |  | **12.22** | **± 1.52** | **N/A** |  |
|  |  |  |  |  |  |  |
| **age at recruitment (yr)** | **32.02** | **± 5.98** | **13.01** | **± 1.48** | **33.96** | **± 3.13** |
|  |  |  |  |  |  |  |
| **Glucose (mg/dl) at recruitment** | **87.2** | **± 3.92** | **171.27** | **±21.65** | **81.5** | **± 4.86** |
|  |  |  |  |  |  |  |
| **Insulin requirement (U/kg/day)** | **N/A** |  | **0.37** | **± 0.05** | **N/A** |  |
|  |  |  |  |  |  |  |
| **HbA1c (%)** | **NA** |  | **7.24** | **± 0.43** | **NA** |  |
|  |  |  |  |  |  |  |

N/A – not applicable

NA – not available
